# Gloss and Speed Judgments Yield Different Fine Tuning of Saccadic Sampling in Dynamic Scenes

**DOI:** 10.1177/2041669519889070

**Published:** 2019-12-15

**Authors:** Matteo Toscani, Ezgi I. Yücel, Katja Doerschner

**Affiliations:** Department of Psychology, Giessen University, Germany; Department of Psychology, University of Washington, Seattle, WA, USA; Department of Psychology, Giessen University, Germany; Department of Psychology & National Magnetic Resonance Research Center, Bilkent University, Turkey

**Keywords:** eye movements, motion, optic flow, surfaces/materials

## Abstract

Image motion contains potential cues about the material properties of objects. In earlier work, we proposed motion cues that could predict whether a moving object would be perceived as shiny or matte. However, whether the visual system uses these cues is still uncertain. Herein, we use the tracking of eye movements as a tool to understand what visual information observers use when engaged in material perception. Observers judged either the gloss or the speed of moving *blobby* shapes in an eye tracking experiment. Results indicate that during glossiness judgments, participants tend to look at gloss-diagnostic dynamic features more than during speed judgments. This suggests a fine tuning of the visual system to properties of moving stimuli: Task relevant information is actively singled out and processed in a dynamically changing environment.

## Introduction

Saccadic eye movements bring different parts of the visual environment onto central vision, so that it can be analyzed in further detail. This strategy maximizes information gain, for example, during visual search (e.g., Najemnik & Geisler, 2005), and minimizes local uncertainty, for example, during shape recognition (e.g., Renninger, Verghese, & Coughlan, 2007). Eye movements are influenced not only by stimulus saliency (as in these examples) but also by task demands. In fact, there is a large body of evidence showing that based on the specific patterns of eye movements, it is possible to identify which task an observer was involved with (for a review, see Boisvert & Bruce, 2016), and visual sampling strategies seem to be optimized for providing information to guide our actions (e.g., Hayhoe, Shrivastava, Mruczek, & Pelz, 2003; Land, Mennie, & Rusted, 1999; for a review, see Hayhoe & Ballard, 2005): For example, when observers move in a virtual reality environment, fixations tend to land on different regions of identical objects, depending on whether participants were asked to approach or avoid the object (Rothkopf, Ballard, & Hayhoe, 2007). Similarly, when looking at pictures, observers fixated different regions of natural objects depending on whether they were asked to categorize, mimic to open, lift, or use them (e.g., Belardinelli, Barabas, Himmelbach, & Butz, 2016; Belardinelli, Herbort, & Butz, 2015). Thus, tracking eye movements can provide insights as to what visual information observers might use when engaged in different perceptual tasks.

In a recent work from our group, we used this approach to show that the maximum luminance is the most diagnostic value for reflectance difference of an object’s luminance distribution and that observers use this feature when judging the lightness of surfaces (Toscani, Valsecchi, & Gegenfurtner, 2013b, 2017). We initially speculated that observers would base their lightness judgments on the brightest regions of the targets because these regions provide an optimal estimate for the surfaces’ reflectance (Adelson, 2000; Gilchrist, 2006), and such a strategy could serve as a heuristic to achieve a stable estimate of lightness independent of knowledge about scene geometry, shape, or illumination. Interestingly, we found this heuristic to vary with the properties of the stimulus (Toscani, Valsecchi, & Gegenufurtner, 2013c): For glossy surfaces, observers tended to fixate not the brightest region (i.e., the highlight) but instead the regions directly adjacent to the specular highlight (Toscani, Valsecchi, & Gegenfurtner, 2013a). This strategy makes, in fact, perfect sense because specular reflections are not diagnostic for an object’s surface color and lightness: The color and intensity of a specular highlight depend, to a large extent, on the illumination properties rather than on the surface reflectance (albedo or color). Thus, the strategy that we found the visual system to use for sampling was optimized for the task at hand and the objects’ properties, focusing on regions of objects which contain the most task-relevant information. What might be the mechanism behind this kind of optimization? An object might first be identified in peripheral vision to roughly estimate its properties (e.g., its overall shape or surface reflectance category). This initial analysis may guide subsequent fixations to the most informative regions and more fine-grained analysis. Such a sequential process could be particularly challenging for the visual system when analyzing dynamic scenes where task relevant information can change (position and quality) over time, yet most of the visual information we encounter is changing dynamically. In this experiment, we investigate how observers’ sampling strategies vary with the demands of the perceptual task in dynamic scenes.

Specifically, we track eye movements in order to investigate whether observers use material-specific motion cues when judging whether an object is glossy or not (Doerschner et al., 2011). Doerschner et al. (2011) proposed three motion cues (optic flow divergence, coverage, and three-dimensional [3D] shape reliability) that could predict whether a moving object would be perceived as shiny or matte. If these cues are, in fact, used by the visual system, observers should look at regions where the cues are prevailing or are particular diagnostic (e.g., regions of high divergence in the optic flow). For visual tasks that involve other judgments, such as perception of speed, a saccadic sampling strategy should maximize other visual cues, such as local motion energy, as speed estimates are based on the pooling of local motion signals (Sekuler, 1992) as sensed by elementary motion detectors (Clifford, Beardsley, & Vaina, 1999). To test whether observers’ sampling strategies vary with the demands of the perceptual task in dynamic scenes, we had observers perform gloss and speed judgments on the same stimuli. The results of our experiment show that in the gloss—but not the speed—judgments task, observers tend to dynamically direct their gaze on the regions where motion cues for glossiness are expressed, that is, the presence of these cues at gaze position in space and time can be used to classify the task. This suggests that the task-dependent sampling strategy of the visual systems goes beyond simply directing attention to different parts of objects or scenes, but that it is also fine-tuned to the dynamic properties of the environment.

## Methods

### Participants

Ten naive observers from the Justus-Liebig University of Giessen volunteered to take part in the experiment. They all had normal or corrected-to-normal visual acuity. All gave written informed consent in accordance with the Code of Ethics of the World Medical Association (Declaration of Helsinki). The experiments were approved by the local ethics committee (approval number LEK 2009-0008).

### Stimuli

**Figure 1.**
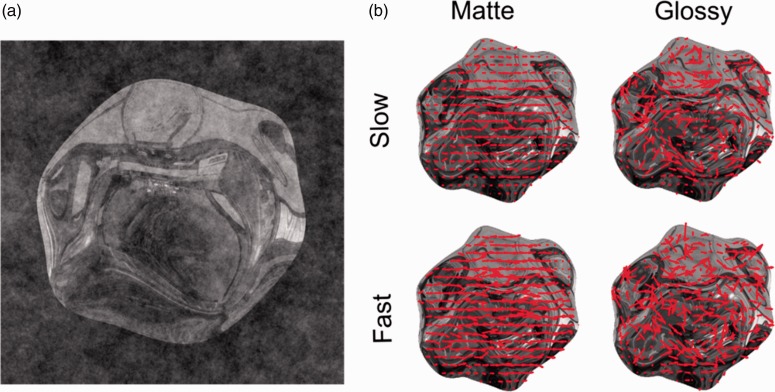
Stimuli. (a) Example shape embedded in noise. (b) Optic flow field for the four classes of stimuli: matte-textured, glossy, fast, and slow. The direction of the arrows indicates the local direction of the flow, the length its energy. In the glossy examples, there is more variability in the local directions, indicating higher divergence than in the matte-textured stimuli. For fast stimuli, the arrows are longer, indicating that these stimuli had higher motion energy. Sample movies are provided in Supplementary Materials.

Stimuli were four 3D shapes, generated by perturbing a unit geosphere primitive (Figure 1(a)) with five sine waves of different orientations and wavelengths. This type of object has been extensively used in material perception (e.g., Adams, Kerrigan, & Graf, 2016; Cholewiak & Fleming, 2013; Cholewiak, Kunsberg, Zucker, & Fleming, 2014; Cholewiak, Vergne, Kunsberg, Zucker, & Fleming, 2015; Doerschner et al., 2011; Fleming, Torralba, & Adelson, 2004; Muryy, Fleming, & Welchman, 2016; Muryy, Welchman, Blake, & Fleming, 2013; Norman, Todd, & Orban, 2004; Toscani et al., 2017).

All objects were illuminated by the Ueno-Shrine Lightprobe (Debevec, 1998) and rendered using the software Gratin (Vergne & Barla, 2015). For each shape, we generated rotations around the vertical axis at a speed of 0.067 degrees per frame in the *slow* condition and 0.134 degrees per frame in the *fast* condition (for 270 frames in total).

Shapes were rendered either as a mirror reflecting surface or were diffusely reflecting and textured. The latter ones were generated by *sticking* a specular reflection pattern to the object’s surface, so that for any frame in the motion sequence, the patterns on the surface would be consistent with specular reflections of the surrounding environment (looking shiny). However, when viewed in motion, the patterns moved with the surface, as if they were painted on, eliciting the percept of a matte surface (Doerschner et al., 2011; Yilmaz & Doerschner, 2014).

In order to prevent a potential ceiling effect in the tasks described here, all images were contrast reduced (by a factor of 2) and embedded in Brownian noise with 1/f^2 spectrum.

### Apparatus

We used the psychtoolbox-3 software (Kleiner et al., 2007) working on MATLAB (http://www.mathworks.com) to display the rendered movies on an Eizo CG223W 10 bit LCD monitor. We linearized the monitor according to standard methods (e.g., Hansen & Gegenfurtner, 2013).

### Procedure and Task

Participants sat in a dark room, with their heads stabilized by a chinrest with 38 cm distance between forehead and the center of the screen. This specific distance was chosen in order to produce large retinal projections of the stimuli, which was needed to reveal potential systematic local differences in gaze allocation (similar to Toscani, Zdravković, & Gegenfurtner, 2016). To familiarize participants with the tasks, the experiment begun with a short demonstration. Two object pairs were presented in a 2 × 2 arrangement around the center of the screen, and observers were asked to indicate which of the two pairs (top or bottom) contained objects with different rotation speeds (in speed task demonstration) or to indicate which of the two pairs has objects made of different materials (in the gloss task demonstration). The stimuli for these demonstrations were the same as those in the experiment, but the images were not degraded by a dynamic noise pattern.

After the demonstration, the eye tracker was calibrated (see section “Eye tracking procedure”). The experiment was separated into two blocks: one for speed and one for gloss judgments. Each block consisted of 16 trials (4 objects × 2 rotation speeds × 2 materials). Each trial started with a fixation on the center of the screen where the eye tracker calibration was checked and if necessary repeated. After a key press from the participant, the stimulus appeared in one of the four possible locations (four corners of the screen) selected at chance, so that participants had to actively shift their gaze from the center of the screen towards the stimulus. Each motion sequence lasted 4.5 seconds (270 frames). Note that during the first and last 10 frames of the animations, the stimulus faded in and out, respectively. We found in pilot trials that this fading made the stimulus appearance more pleasant and caused less strain to the eye. Participants indicated via button press the speed (fast or slow, speed block) or the glossiness (glossy or matte, material block). They were instructed to free their gaze during trials after the fixation period.

### Eye Tracking Procedure

Gaze position signals were recorded with a head mounted eye tracker (EyeLink II; SR Research, Ottawa, ON), sampling at 500 Hz. At the beginning of each experiment, the eye tracking system was calibrated. If the validation procedure revealed a mean error bigger than 0.4° visual angle, the calibration was repeated. At the beginning of each trial, the calibration was reexamined. If the error was more than 1° visual angle, a new calibration was performed; otherwise, a drift correction was applied.

### Analyses

We defined two predictors that would entail the information necessary to perform the respective tasks. We first computed optic flow for each frame of our image sequences (Doerschner et al., 2011; Gautama & Van Hulle, 2002). We chose motion *energy*, computed as the norm of the optic flow field vectors, as a candidate predictor for speed judgments. Figure 1(b) shows the optic flow field for one frame for each of the four classes of stimuli, and here *energy* corresponds to the length of the vectors (i.e., red arrows): Fast stimuli have higher *energy*. Following our previous work (Doerschner et al., 2011), we choose *divergence* as a candidate predictor for gloss judgments. We singled out this measure (Doerschner et al., 2011; Yilmaz & Doerschner, 2014) because it is intuitive to understand and easy to implement. Essentially, it provides a measure of how much the direction of vectors in the flow field locally deviate from one to another. For a matte, rotating (around the vertical axis) shape, texture patterns move together (more or less homogenously) with the surface to which they are attached and there is little divergence in the optic flow. Conversely, a specular object generates much higher divergence in the flow field, as specular flow does not depend primarily on the object motion but on the undulations of the 3D surface (i.e., its curvature; see Dovencioglu, Ben-Shahar, Barla, & Doerschner, 2017; Koenderink & van Doorn, 1980). This is also illustrated in Figure 1(b), that is, the direction of the optic flow, as indicated by the red arrows, is more uniform in the matte than in the glossy stimuli. As we were not interested in the direction of divergence, but rather its magnitude, we use the latter (i.e., the absolute value of divergence) in the following analyses.

In a subsequent step, we tested whether these predictors could discriminate between our stimulus classes. In our previous work, divergence was computed over the entire frame (Doerschner et al., 2011); here, we were interested in the information surrounding the gaze position on the image. Thus, we ran a simulation to test whether local information from randomly chosen small circular portions of our stimuli (∼1.5° of visual angle radius) were enough to tell apart two classes of stimuli (glossy vs. matte & fast vs. slow) based on *energy* and *divergence.* We sampled 100 circular patches for each stimulus class, randomizing the position in space and time. For each of the patches, we averaged local *energy* and *divergence*. Then, we ran a linear classification analysis to test whether samples from the different stimulus classes could be linearly separated based on the predictors. We trained the classifier with a leave-one-out procedure to prevent for overfitting (Milojevic, Ennis, Toscani, & Gegenfurtner, 2018; Toscani et al., 2017; Wiebel, Toscani, & Gegenfurtner, 2015).

Lastly, we used logistic regression to predict the task (speed or gloss judgments) based on local information at gaze position. Specifically, we related each gaze sample to its corresponding stimulus frame in time and we extracted local *energy* and *divergence* within a circle surrounding the gaze position on the image (∼1.5° of visual angle radius), then we averaged across the circle, and then, across trials. Thus, for each trial, we had one value for *energy* and one for *divergence*. Additional independent variables were *speed* (slow vs. fast) and *gloss* (matte vs. gloss). The dependent variable was *probability of the task involving speed judgments* (vs. *gloss judgments*). For each stimulus class, we *z*-transformed *energy* and *divergence* so that results could not be driven by stimulus difference, and regression coefficients would be expressed in the same unit (beta weights). We compared a full model with all the interaction terms, and a nested model with no interactions. Logistic regression models were fit individually for each observer, and the regression coefficients tested against the null hypothesis of being equal to zero in the population.

## Results

**Figure 2.**
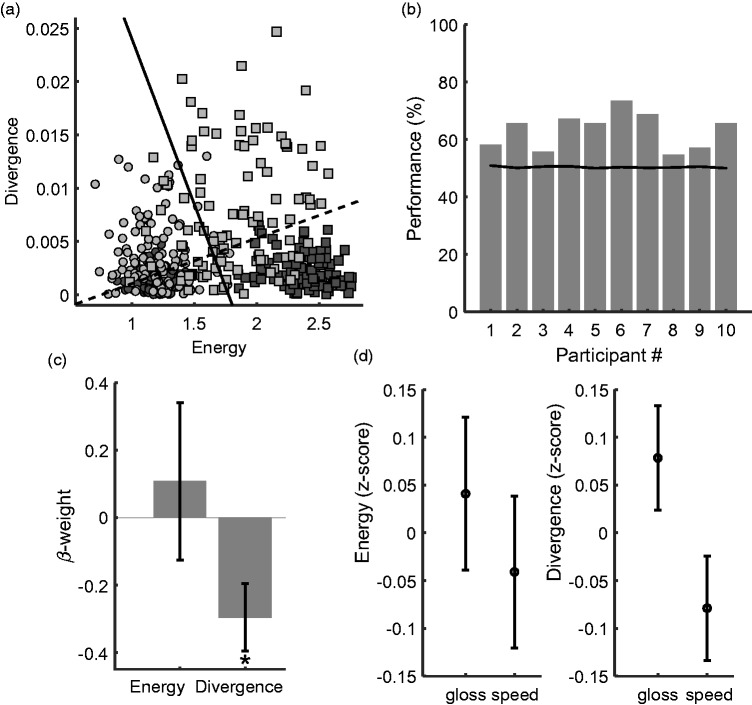
Results. (a) Simulation. Motion energy on the *x*-axis and divergence on the *y*-axis. Squares indicate samples from fast stimuli and circles from slow stimuli. Light gray denotes glossy stimuli, and dark gray denotes matte stimuli. The boundary for speed classification is indicated by the continuous black line; the boundary for gloss classification is indicated by the dashed line. (b) Model performance for each participant. (c) Beta weights for the four predictors of the logistic regression model averaged across observers. Error bars are one standard error of the mean. (d) Energy (left panel) and divergence (right panel) at gaze position in gloss and speed tasks.

Figure 2(a) shows *energy* and *divergence* values for each of the 100 samples for each stimulus class. Fast (squares) exhibit higher local *energy* than slow (circles) stimuli. *Divergence* tends to be higher for glossy (light gray circles and squares) than for matte-textured stimuli (dark gray). Classification analysis revealed that by means of both *divergence* and *energy*, it is possible to discriminate between slow and fast stimuli with 92% accuracy and between matte and glossy stimuli with 76% accuracy. Speed classification (slow vs. fast) seemed dominated by *energy* whereas gloss (matte vs glossy) classification by *divergence. Energy* alone could classify speed with 91% accuracy; *divergence* alone could classify gloss with 71% accuracy.

We used the Akaike Information Criterion to compare the full model (with the all the interaction terms) and the nested model with no interactions, the latter of which we selected for further analyses. The model, fitted separately for each observer, could predict the task, based on *energy* and *divergence* better than chance (Figure 2(b)). The empirical chance level was computed using a bootstrapping procedure as follows: The regression model was fit to the data after randomizing the correspondence between information at gaze position and task, and then the fitted model was used to predict the task based on *energy* and *divergence* at gaze position. This procedure was repeated 500 times, and the performance was averaged across iterations. Empirical chance level (Figure 2(b), dashed black line) was found to be around 50% for all observers. A one-sample *t*-test revealed a significant difference between the performance of the model and the empirical chance level, *t*(9)=6.391, *p* < .001, two-tailed. As the model could predict the data better than chance, we were able to interpret its coefficients (Figure 2(c)). The negative coefficient associated with *divergence* indicates that in the speed judgment task, observers tended to look less at local divergence than in the gloss judgment task. A one-sample *t*-test (two-tailed) confirms that the beta weight associated with *divergence* is on average lower than zero, *t*(9)= –3, *p* < .05). In fact, divergence at gaze position was higher in the gloss discrimination task than in the speed discrimination task (Figure 2(d)). We did not find a significance effect of *energy*, *speed*, or *gloss* on gaze position.

However, for all tasks and stimuli, people tended to fixate on regions of higher energy, that is, 0.39 *SD*s compared to stimulus average, *t*(9)= 20.04, *p* < .0001.

## Summary and Discussion

We investigated whether observers would look more at gloss-diagnostic dynamic features when they judge the glossiness of rotating, 3D objects, than when engaged in other perceptual tasks. Indeed, we found that participants tended to look at regions of high divergence in the optic flow more during gloss judgments, than when judging the speed of the same set of stimuli. Such a strategy would be consistent with the idea that the visual system is not systematically sampling all the perceptually relevant stimulus properties to represent them as a whole in memory, but that instead, it is postponing the gathering of *task-relevant* information until just before it is required (*just-in-time* strategy), presumably to reduce the memory load (Ballard, Heyhoe, & Pelz, 1995). This sampling *strategy* appears to be hold for both action planning and perceptual tasks (as in our experiment).

Interestingly, our results also suggest that—regardless of the task—in dynamic scenes, participants tend to always look at regions of higher motion energy. Taken together, these findings might suggest that eye-movement patterns in our experiment reflect the involvement of two cortical mechanisms: a low-level mechanism driven primarily by motion energy of the stimulus (present in all tasks), and a high-level mechanism driven by specific task demands (e.g., judgments of material qualities) and higher order stimulus properties (like optic flow divergence).
